# Biological features of squamous cell carcinoma of the anus—a deep dive review

**DOI:** 10.1016/j.esmogo.2025.100157

**Published:** 2025-04-03

**Authors:** R.J. Samuel, N. Winder, M. Busk, K. Lycke Wind, G. Guerra, A.G. Heriot, D.C. Gilbert, A. Macdonald, K.-L. Garm Spindler

**Affiliations:** 1 Leeds Institute for Medical Research, University of Leeds, Leeds, UK; 2 Division of Cancer Sciences, School of Medical Science, University of Manchester, Manchester, UK; 3 School of Molecular and Cellular Biology, Faculty of Biological Sciences, University of Leeds, Leeds, UK; 4 Department of Experimental Clinical Oncology, Aarhus University Hospital, Aarhus, Denmark; 5 Danish Center for Particle Therapy, Aarhus University Hospital, Aarhus, Denmark; 6 Division of Cancer Surgery, Peter MacCallum Cancer Centre, Melbourne, Australia; 7 Department of Surgery, St Vincent’s Hospital, The University of Melbourne, Melbourne, Australia; 8 The Sir Peter MacCallum Department of Oncology, The University of Melbourne, Melbourne, Australia; 9 MRC Clinical Trials Unit at UCL, London, UK; 10 Sussex Cancer Centre, University Hospitals Sussex NHS Foundation Trust, Royal Sussex County Hospital, Brighton, UK

**Keywords:** anal cancer, radiotherapy, HPV, hypoxia, immunotherapy

## Abstract

Squamous cell carcinoma of the anus (SCCA) is a human papillomavirus (HPV)-associated cancer with an increasing incidence. Localized disease is treated curatively with chemoradiotherapy (CRT) but has substantial toxicities and impact on quality of life. Despite increasing research over the past decade, much of our biological understanding of SCCA is extrapolated from other HPV-associated cancers. Recently launched initiatives aim to connect clinicians and basic researchers working in SCCA to foster collaboration and knowledge sharing and facilitate access to SCCA materials. To improve outcomes for SCCA patients, a better understanding of SCCA-specific biology is required. This review will discuss areas of SCCA biology that are important to developing targets for drug development and biomarker-based individualized precision treatment. It will discuss the current understanding of radiosensitivity, differences in HPV-positive versus HPV-negative disease, hypoxia, immune environment, molecular characteristics, circulating tumor HPV-DNA, and the microbiome. It will detail when this understanding has been extrapolated from other HPV-associated cancers. Finally, it will discuss our current and upcoming preclinical biological models and discuss future models needed to further our understanding of SCCA biology.

## Highlights

• Anal cancer is rare but increasing and associated with HPV.
• Most biological understanding is extrapolated from other HPV-associated cancers.
• There is an increasing number of anal cancer specific biological models.
• To improve outcomes for patients, more specific biological research is needed.

## Graphical abstract

Graphical abstract

**Figure:**
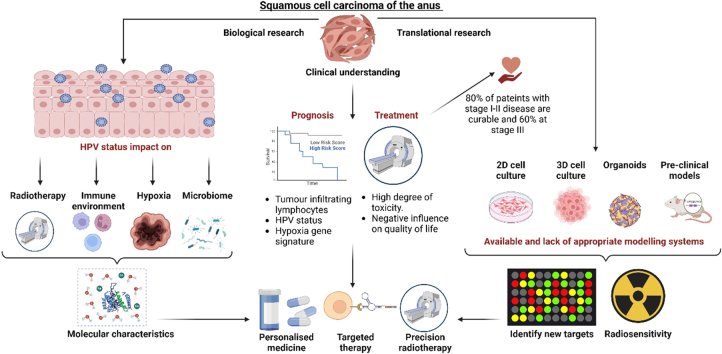

## Introduction

Squamous cell carcinomas (SCCs) are frequent cancers from the stratified squamous epithelium characterized by a continuous protective surface. The epithelium fully regenerates over a few weeks and has a unique ability to withstand external trauma and abrasions. Furthermore, it can accelerate its repopulation and maintain function during hypoxic conditions. When treated with radiotherapy (RT), SCCs express a steep dose-response relation and show the ability for accelerated repopulation.^1^

Squamous cell carcinoma of the anus (SCCA) is a rare disease with an increasing incidence. Primary treatment of localized disease is chemoradiotherapy (CRT), which is delivered to the anal canal as well as the pelvic and inguinal lymph node areas.^2^ Prognosis is heavily dictated by stage at presentation, with national cohorts reporting 3-year disease-free survival (DFS) of 80%-85% and 65%-70% for early and advanced localized disease, respectively, with 3-year overall survival (OS) for metastatic disease of ∼20%.^3^^,^^4^ Although improvements in RT technique have reduced toxicities, there is still a high degree of severe acute and late toxicity with negative influence on quality of life.^5^^,^^6^ SCCA is an often-overlooked disease due to its rarity and complex nature. Both disease and treatment can be associated with tabu and loneliness for patients. Although there has been increasing clinical research activity during the past decade, biological knowledge is lacking and often extrapolated from other SCCs. Deeper biological knowledge is needed to enhance our understanding of radiosensitivity, targets for drug development, and biomarker-based individualized precision treatment.^7^ Recent initiatives from the International Rare Cancers Initiative (IRCI) Anal Cancer Group and the International Multidisciplinary Anal Cancer Consortium (IMAAC) focus on connecting clinicians and basic researchers to collaborate, share knowledge and SCCA materials.^8^^,^^9^ This review aims to provide a status of current basic biological and translational research with a specific focus on the role of human papillomavirus (HPV) status, radiosensitivity and hypoxia, molecular characteristics, and the immune environment. It will discuss our current preclinical biological models and those needed to further understand SCCA biology. With this deep dive, we hope to facilitate multidisciplinary discussions for future focus on SCCA research within an international context.

### Epidemiology and the role of HPV in SCCA

**Figure 1:**
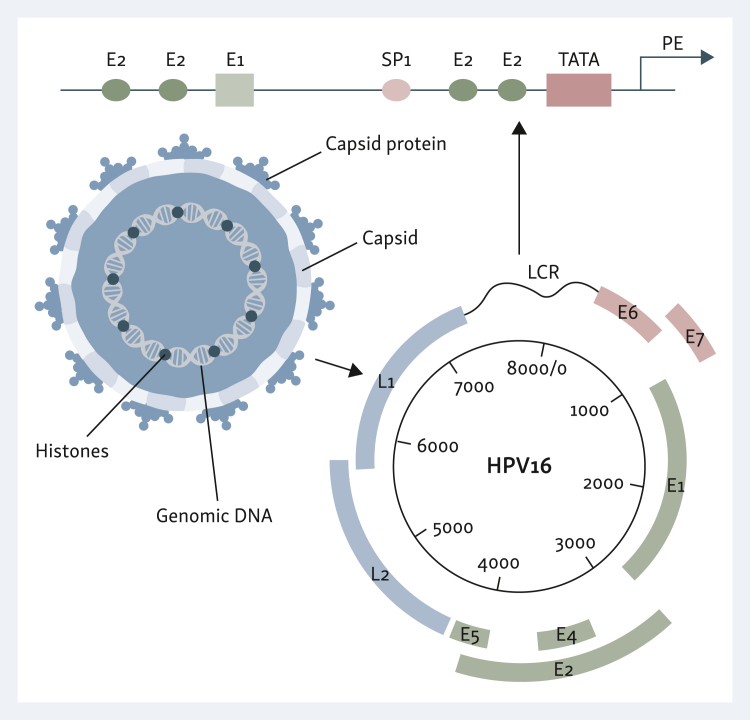
Structural architecture of HPV, alongside the organization of the HPV genome and sequence of viral gene expression. HPV, human papillomavirus.

There were an estimated 54 000 cases of SCCA in 2022, with global trends showing annual increases in incidence and mortality.^10^^,^^11^ HPV is a small (50-80 nm), non-enveloped virus with a circular double-stranded DNA (dsDNA) genome 8 kb in length (Figure 1).^12^ HPV infections are thought to occur predominantly through skin-skin or skin-mucosa interaction, with sexual transmission being the most documented cause. HPV enters the host directly through the epidermis and mucous membranes. Approximately 85% of individuals who are HPV-positive will be asymptomatic on initial infection; however, the remaining 15% experience symptoms such as skin lesions/warts. Reoccurring or persistent infection, especially with high-risk HPV (HR-HPV) types, can lead to cellular dysplasia and eventually cancer. The proportion of HPV-attributable cancer at each of the anatomical sites varies, being as high as 99.7% for cervical cancer (CC) to about 2% for oral cavity cancers.^13^^,^^14^

At least 90% of SCCAs are HPV-associated with p16INK4a (p16) immunohistochemistry used to infer HPV causation clinically. High-sensitivity genotyping suggests this figure may be higher and closer to the frequency seen in CC.^14^ HPV16 is the most common HR-HPV strain, accounting for about 55% of SCCA, followed by HPV18 (15%), with HPV31, 33, 45, 52, and 58 accounting for a combined additional 17%.^13^

The introduction of the HPV quadrivalent (hr-HPV 6/11/16/18) vaccination, first approved in 2006, is already having an early impact on cancer incidence in countries with established vaccination programs. The HPV vaccination program in England initiated in 2008 and, by mid-2020, had prevented an estimated 687 CC cancer cases (95% confidence interval 556 to 819 cases) and 23 192 (22 163 to 24 220) cases of CIN3s.^15^ The early impact on CC is linked to its younger age of incidence compared with other HPV-associated cancer, and a reduction in SCCA is not expected for another 20-30 years. The COVID-19 pandemic led to a decline in HPV vaccination rates and to achieve the most significant reduction in HPV-associated cancers including SCCA, further research is needed to understand the reasons behind vaccine hesitancy.^16^^,^^17^

### Biology and radiobiological features of HPV-positive and -negative disease

RT is the main curative treatment of locally advanced SCCs. Regardless of HPV status, squamous cells’ ability to rapidly repopulate after injury allows for accelerated repopulation after RT and is an essential feature in radioresistance. This is underbuilt by data suggesting that unscheduled treatment interruptions that increase overall treatment time negatively impact outcomes.^18^ In contrast to other HPV-associated SCCs, hyperfractionated RT regimens have not been investigated for SCCA.

Across these cancers, HPV-negative cancer has a worse prognosis than HPV-positive cancer, possibly due to differences in response to RT. Much of the data on the differences in biology between HPV-positive and HPV-negative disease has been derived from CC and head and neck cancer (HNSCC). Upon HPV genome integration, many of the signaling pathways of normal DNA repair mechanisms, such as ataxia telangiectasia-mutated (ATM) and Rad3-related (ATR), are affected, allowing continual increasing G2/M cycle arrest.^19^ Data from CC shows that DNA damage generated from direct or indirect ionization enables permanent damage within the cells due to their compromised ability to repair dsDNA breaks, leading to increased radiosensitivity.^20^ In HNSCC cell lines, the survival fraction after 2 Gy (SF2) is 0.22 for HPV-positive versus 0.59 for HPV-negative.^21^ In addition, the HPV-positive tumor microenvironment (TME) is characterized as more pro-inflammatory after RT, which may enhance its antitumor effects.^22^

**Figure 2:**
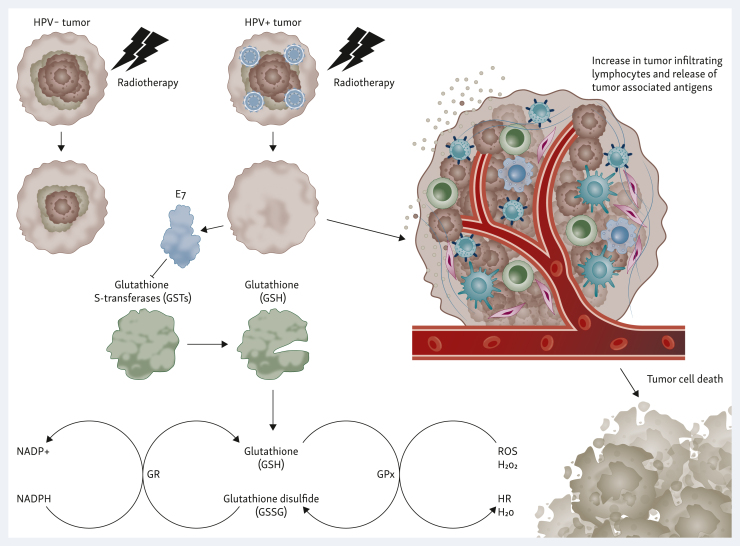
**How HPV infection generates radiosensitive tumors.** E7 viral oncoprotein can inhibit the function of detoxification enzymes, preventing the removal of free radicals and increasing the reactive oxygen species within the tumor, leading to tumor cell death. HPV, human papillomavirus.

Impaired DNA repair mechanisms resulting from HPV manipulation of signaling pathways can also influence radiosensitivity by altering the metabolic activity within tumors, which can reduce tumor hypoxia and resident cancer stem cells (CSCs). HPV-negative HNSCC cancers show a 49% increase in resident CSCs compared with HPV-positive cancer, resulting in a reduced cellular immune response.^23^ CSCs are usually located within hypoxic areas of tumors and have been shown to enhance repair of DNA damage more effectively in HPV-negative tumors compared with HPV-positive tumors, which contain more differentiated tumor cells.^24^ RT efficacy heavily relies on oxygenated areas to enable the formation of oxygen free radicals. HPV’s host cell transformation can enhance DNA damage from RT through viral oncoproteins E6 and E7. E6 can reduce the number of antioxidants within the TME, while E7 inhibits glutathione transferase, a protein the function of which is to scavenge free radicals, enabling the accumulation of reactive oxygen species (Figure 2).^12^^,^^25^

HPV-negative disease can occur through spontaneous mutations, exposure to certain stimuli (e.g. smoking), and hereditary conditions.^26^^,^^27^ In HPV-negative HNSCC, carcinogenic nitrosamines generated from tobacco covalently bind to DNA, forming DNA adducts producing dsDNA breaks leading to hypermutations and chromosomal instability. This in turn dysregulates homeostatic pathways within the cells, such as the fibroblast growth factor receptors (FGFRs) and epithelial growth factor receptor signaling which subsequently affect RAS/RAF/ERK, PI3K, and JAK/STAT pathways, resulting in uncontrolled cellular proliferation.^28^^,^^29^ Smoking is a risk factor for HPV-negative SCCA and it can be extrapolated that similar pathways are responsible in HPV-negative SCCA.^30^^,^^31^

HPV-positive and HPV-negative cancers are immunologically distinct, with HPV-negative CC, SCCA, and HNSCC having lower tumor-infiltrating lymphocytes (TILs).^32^, ^33^, ^34^ Additionally, activation/exhaustion markers on B, T, and NK lymphocytes and T-cell receptor diversity are significantly lower in HPV-negative tumors.^35^ After RT, HPV-positive tumors release greater quantities of tumor-associated antigens, stimulating a greater pro-inflammatory, antitumor response by increasing infiltrating T cells alongside immune regulatory proteins and chemokines.^36^ Integration of HPV E6/E7 into the host genome leads to their deregulated expression and further inactivation of the tumor suppressors p53 and pRB, respectively, preventing cell cycle arrest and reducing sensitivity to apoptotic signaling.^29^

### The microbiome

Preliminary data suggest that the microbiome may relate to the severity of toxicity after CRT for SCCA, but differences between HPV-positive and HPV-negative microbiome for SCCA are yet to be identified.^37^ A study in HNSCC reported that *Legionella*, *Megasphaera*, and *Anaerolineae* species were abundant in HPV-positive HNSCC compared with HPV-negative HNSCC.^38^ These differences were associated with response to therapy and seem to influence the disease’s evolution, thereby underlining the potential importance of the microbiome in HPV-associated cancers. More specific studies in SCCA, however, are needed.

### The role of hypoxia in SCCA

Hypoxia is a well-accepted feature of the TME and is associated with radioresistance.^39^ Tumor hypoxia is clinically relevant as it can be quantified and modified. Over the years, different methods to estimate tumor hypoxia have been investigated including direct measurement of oxygen tension in the tumor, visualization by imaging using hypoxia-specific tracers, or measurement of endogenous markers of hypoxia (e.g. up-regulation of hypoxia-related genes).^40^ To overcome the radioresistant effect of hypoxia, various hypoxia modification strategies have been explored such as inhalation of oxygen-enriched gas, blood transfusion in the case of low pretreatment hemoglobin or treatment with hypoxic cell radiosensitizer in combination with RT.^41^ Hypoxia has been extensively studied in HNSCC where hypoxic modification has been shown to reduce locoregional failure (LRF), DFS, and OS.^42^ Most treatment failures in SCCA are locoregional indicating that radioresistance is the main cause of treatment failure.^43^

Nevertheless, the investigation of hypoxia in SCCA is sparse and is limited to only a few studies. One study has investigated the use of oxygen-enhanced magnetic resonance imaging as an indication of tumor perfusion during CRT.^44^ It only included a small number of patients and therefore could not correlate results to clinical outcomes. In another study, the expression of hypoxia-related genes has been evaluated in 79 SCCA tumor tissue samples. This study evaluated hypoxia using a 15-gene hypoxia classifier adopted from HNSCC and classified them as either more or less hypoxic.^45^ The study could not report a significant difference in local control between the more and less hypoxic group, but the limited sample size, the relatively short follow-up time, and the low number of events limit conclusions. The same 15-gene hypoxia classifier has been used in a larger sample size of 368 tumor samples. The classification resulted in significantly higher LRF in patients with more hypoxic tumors compared with patients with less hypoxic tumors (unpublished, presented at IMAAC2023). Hypoxia could play an important role in future studies in SCCA to identify patients requiring treatment intensification and improve locoregional control.

### The importance of the immune environment in SCCA

A higher incidence in people with human immunodeficiency virus and solid organ transplant recipients empirically shows the importance of the immune environment for SCCA.^46^ Elevated neutrophil–lymphocyte ratio, neutropenia, and leukocytosis have all been found to be negatively prognostic for SCCA and proposed as potential biomarkers for SCCA, highlighting the importance of the host immune response even without significant pharmacological or pathological immunosuppression.^47^^,^^48^ Furthermore, since it can take up to 6 months after finishing CRT to reach a complete response in SCCA, a degree of immune clearance is likely needed to achieve a complete response.^49^ Failure of immune clearance may explain some LRFs in the first few months after CRT, indicating that there could be a window of opportunity to intervene and improve outcomes.

Viral oncoproteins E6 and E7 are tumor-specific antigens that drive TILs into HPV-positive cancers. CD8-positive TILs from CC and HNSCC cells can be stimulated using HPV antigens, but this analysis has not been carried out in SCCA.^50^^,^^51^ In SCCA, the prognostic value of TILs was demonstrated in two combined cohorts of patients from the UK and Denmark, with TILs classified into high, medium, or low.^32^ It showed that the TILs score added to p16 status for the prognostication of SCCA patients receiving CRT. The complexity of the combined feature of HPV status and TILs highlights the importance of a deeper understanding of the immune environment in SCCA.

Reduction of expression of MHC molecules, a skew towards a Th2 CD4-positive response, and inhibition of cGAS-STING have all been proposed as mechanisms of immune evasion in HPV-positive CC and HNSCC, but there are currently no SCCA-specific data on HPV-associated immune evasion mechanisms.^52^ The programmed cell death protein 1 (PD-1)/programmed death-ligand 1 (PD-L1) axis is the mechanism of immune evasion in some cancers and is used as a biomarker for response to immunotherapy.^53^ Depending on the study, 50%-65% of SCCAs express PD-L1 and most find high PD-L1 correlates with improved survival after standard CRT. Differences in scoring systems and staining, however, still make results challenging to interpret.^54^, ^55^, ^56^ In localized disease improved outcomes in SCCA have been associated with both higher HPV viral load and high PD-1 expression, but there are conflicting data on the correlation between high viral load and PD-L1 expression.^57^, ^58^, ^59^, ^60^

Microsatellite instability, deficient mismatch repair (d-MMR), and high tumor mutational burden (H-TMB) (>10 mutations/megabase) are associated biological features that are signs of high genomic instability. These are identified as markers for response to immunotherapy in other cancers. The translational analysis from the CARACAS study showed that out of 40 available cases, 5 had H-TMB, only 1 of these d-MMR, and 10 from the available 52 cases expressed PD-L1 (combined positive score >40).^61^ This low frequency indicates that viral antigens rather than genomic instability drive the host immune response in HPV-positive SCCA.

Results from trials using checkpoint inhibitors as second-line treatment of metastatic SCCA have had variable results, with objective response rates ranging from 9% to 27% across several trials.^62^ Selected patients from these trials have had sustained responses of up to 8 years. Although there is a trend for better response in PD-L1-positive cancers in some trials, it is agreed that the current biomarkers cannot adequately identify patients most likely to benefit and further translational studies are needed.

The results of the international phase III POD1UM/InterAACT2 trial were reported at ESMO2024.^63^ It compared retifanlimab, a PD-1 antibody, in combination with standard of care carboplatin–paclitaxel as first-line treatment of inoperable locally recurrent/metastatic SCCA with standard of care. The trial met its primary endpoint, with significantly higher progression-free survival in patients who received retifanlimab (9.3 versus 7.39 months, hazard ratio [95% confidence interval 0.63 (0.47-0.84), *P* = 0.0006]. This is an exciting result that will likely lead to a change in the standard of care for this patient cohort. Results of the translational analysis are eagerly awaited.

Other areas of interest in SCCA immunotherapy have emerged. High indoleamine 2,3-dioxygenase 1 (IDO1) was found to be a poor prognostic marker, and a trial of IDO1-targeted therapy was suggested.^64^ Metastatic HPV-positive SCCA patients have been included in various tumor-agnostic trials for HPV-positive cancer, such as bintrafusp alfa, a bifunctional fusion protein targeting transforming growth factor-β and PD-L1, and more recently messenger RNA vaccine trials, but results for these are awaited.^65^^,^^66^

Multiple trials combine checkpoint inhibitors with RT for SCCA, although early results from most RT-immunotherapy (RT-IO) trials in HPV-associated cancers have failed.^67^, ^68^, ^69^, ^70^, ^71^ Optimal RT fields, choice and schedules of immunotherapeutic drug(s), and patient selection are still to be identified.^72^ Integrating biological studies and translational research is important for all future RT-IO trials.

### Identified molecular characteristics of SCCA

To develop novel therapeutics for SCCA, a better understanding of its molecular characteristics is required. Mutations in *PIK3CA* and *FBXW7* are the most found in HPV-positive disease, whereas *TP53* and *CDKN2a* are most common in HPV-negative disease. These findings are relatively consistent across different ethnic groups, stages of disease, before CRT versus after CRT, and metastatic and localized disease.^55^^,^^56^^,^^73^ At least two studies have found that mutations in *PIK3CA* are a negative prognostic marker in HPV-positive SCCA after salvage abdominoperineal resection, with *KMT2* mutations a negative prognostic marker in one of these studies.^74^^,^^75^ In contrast, no association has been found between mutation status, including *PIK3CA*, and response to first-line chemotherapy in advanced SCCA.^60^^,^^76^ Further work is required to understand these differences in the prognostic role of mutational status in different patient populations. Epidermal growth factor receptor (EGFR) overexpression is common in SCCA. Anti-EGFR therapies have shown efficacy in metastatic SCCA and potential benefit combined with CRT, but none of these trials stratified for EGFR expression.^77^^,^^78^

Despite recent improvements in molecular characterization, there is currently little precision medicine for SCCA, and no validated biomarkers used to stratify treatment. Even HPV status, the most basic and well-characterized ‘molecular profile’ of SCCA is not used to tailor CRT, although it is currently being investigated in a Canadian trial.^79^

Overall, there is an unmet need for SCCA-specific clinical trials to develop target-specific therapies for future patients, selecting or stratifying for biomarkers with extensive exploratory analysis to maximize learning from each patient. A recent excellent review by Iseas and colleagues comprehensively describes the advances in genomics, epigenomics, transcriptomics, and proteomics in more detail, providing insights for developing SCCA precision medicine.^80^

### Circulating tumor DNA research in SCCA

**Figure 3:**
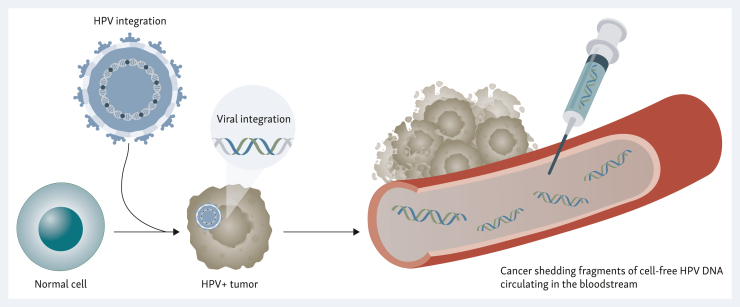
HPV integration into the tumor genome and subsequent cell-free DNA shedding allows HPV DNA to be used as a surrogate for circulating-tumor DNA. HPV, human papillomavirus.

Currently, there are no validated blood-borne biomarkers to assist in clinical decision-making in SCCA, but circulating tumor DNA (ctDNA) is a promising new tool used in various solid tumors.^81^ Small fragments of cell-free DNA circulate in the bloodstream, giving timely information on molecular characteristics, disease status, and prognostic information.^82^ ctDNA can be detected by measuring tumor-specific mutations or epigenetic alterations. Since the elimination half-life of ctDNA is short (<2 h), biological clearance is expected immediately after curative treatment.^83^ Data indicate that the presence of ctDNA in plasma after surgery represents microscopic residual disease and is known to imply an almost 100% risk of recurrence and a poor outcome.^83^ Most ctDNA studies report from CRC and lung cancer, but there is increasing interest in ctDNA analysis in SCCs. Notably, in HPV-associated cancers, HPV can be integrated into the tumor genome and consequently detected in the tumor DNA as a surrogate for ctDNA (Figure 3).^12^^,^^84^ The presence of ctDNA-HPV in SCCA was confirmed in recent studies indicating that up to 93% of cases can be ctDNA-positive depending on tumor stage and analytical method used.^85^ A few studies have shown ctDNA declines during CRT, and residual detectable ctDNA-HPV after CRT seems associated with shorter DFS.^86^ This has led to the initiation of a randomized Nordic trial prospectively investigating ctDNA-guided follow-up.^87^ A single study demonstrated three distinct elimination patterns during CRT and a high risk of failure in patients who did not obtain clearance during treatment.^88^ In metastatic disease, persistent ctDNA-HPV seems associated with poor prognosis after first-line chemotherapy.^89^

There are few published studies (<10), and the total number of patients included is limited. Data support the biological and clinical observations from other solid tumors, however, and collaboration should be encouraged to ensure systematic collection of samples and relevant clinical endpoints.

### Available biological model systems of SCCA

HPV-associated cancers have pioneered biological models for cancer. Cancer cell lines were the first *in vitro* cancer models produced, providing an indefinite source of biological material now fundamental for cancer research. The first human cell line was a CC HPV-positive line, taken from Henrietta Lacks in 1951 (HeLa) and has been integral to many research advances, even being sent into space before manned missions to help understand the effects of radiation on future astronauts.^90^ Since then, at least 52 CC lines and 73 HNSCC lines have been developed.^91^^,^^92^ These cell lines have a vast array of characteristics—they can be HPV-negative or HPV-positive for several HPV subtypes derived from primary or metastatic lesions, have different molecular profiles, and have different chemosensitivities and radiosensitivities. This array of characteristics allows researchers to answer specific scientific questions for CC and HNSCC. In contrast, to the best of our knowledge, the first five SCCA cell lines were developed in 2021.^93^

This panel of cell lines was established from patients with both HPV-positive and HPV-negative SCCA, including primary and relapsed disease. Whole exome sequencing revealed that these lines represent the SCCA patient population. They harbor many of the genomic aberrations frequently identified in SCCA. All lines were found to have strong tumorigenicity in a spectrum of immunocompetent and immunocompromised mice, with the histology of the xenografts demonstrating close recapitulation of the parent tumor. The panel of lines was assessed for cytotoxicity, with the two relapsed lines having substantial resistance to 5-fluorouracil and all lines appearing to display resistance to mitomycin-C. A clonogenic RT assay confirmed the HPV-negative line to be the most radioresistant. A *PI3Kα-*specific inhibitor BYL719 (alpelisib) was also assessed, with sensitivity demonstrated in four cell lines harboring mutations or copy number gains at the *PIK3CA* locus.

The two-dimensional (2D) growth of cell lines does not allow for the nutrient and oxygen gradient seen in tumors. Some of these cell lines can grow in three-dimensional (3D) cultures known as spheroids that replicate this aspect of tumor heterogeneity and better mimic drug responses seen in the clinic. For CC, five cell lines, including HeLa, demonstrated chemotherapy responses closer to tumors for 3D models compared with 2D models of the same cell line, with ADAM17 identified as a novel target to overcome chemotherapy resistance.^94^ For HNSCC, differences in radiobiological response between two HPV-positive and HPV-negative spheroids models allowed for investigation of mechanisms of RT response.^95^ Although technically challenging, immune cells can be added to spheroid cultures, allowing for the assessment of immunotherapy efficacy and has successfully been carried out using CC cell lines.^96^

SCCA tumoroids derived from cell lines have been described, with comparative histological features to that of the patient tumor from which they were derived. The tumoroids demonstrated modulation of PD-L1 expression in the presence of interferon-gamma and three of the tumoroid lines were utilized in patient-matched TIL co-culture cytotoxic assays (unpublished data). This allowed assessment of TIL-directed killing, with the tumoroid line derived from a patient with a primary untreated cancer demonstrating pronounced cell death, compared with the two tumoroid lines derived from relapsed patients. The addition of an anti-PD1 antibody to the assay did not alter the TIL cytotoxic function in one of the relapsed tumoroid lines, despite significant PD-L1 expression.

Patient-derived organoids (PDOs) are another 3D tumor model generated directly from a patient’s tumor tissue. They retain the tumor’s natural cancer cell genetic heterogeneity rather than just oxygen and nutrient gradients, better preserving its pathophysiology. Spontaneously forming 3D structures based on their genetic programming allows for better study of the TME. Numerous studies have been published for CC PDOs, showing their use for precision medicine platforms and identifying novel drug targets.^97^^,^^98^ For HNSCC, PDOs have been developed that could be used for biomarker discovery and validation and assess response to novel therapeutics.^99^^,^^100^ A panel of seven human SCCA PDOs have been established in Australia, which recapitulate the morphological and histological characteristics of the parent tumor, have a genetic profile consistent with SCCA, and are undergoing further characterization that will provide a useful future resource (unpublished).

Traditional xenograft tumor models are established from patient-derived tumor cell lines grown *in vitro*. In contrast, the term ‘patient-derived xenograft’ (PDX) is reserved for models that are established in immunocompromised rodents (typically mice) directly from a patient tumor biopsy or a surgical specimen. A major (theoretical) advantage of PDX models is because they bypass long-term culturing and associated risk for clone selection/enrichment and drift in geno- and phenotypes, they recapitulate the biology of the original tumors more faithfully, including the cellular and TME heterogeneity. Thus, PDX models may provide highly valuable information on patient biology, TME, biomarkers, and treatment sensitivity.^101^^,^^102^

**Figure 4:**
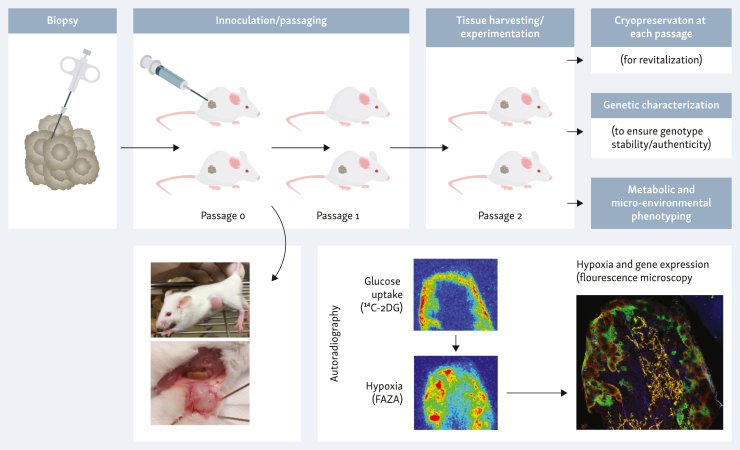
General workflow applied when establishing PDX models, including examples of analyses of TME and metabolism. PDX, patient-derived xenograft; TME, tumor microenvironment.

To the best of our knowledge, very limited work has been done on SCCA. The cell lines described above were used for the first example of an SCCA PDX model. As part of establishing the cell lines, when tumor biopsy material was limited, tumor tissue was initially expanded by implantation of biopsy material intramuscularly into severely immunocompromised mice, followed by the later establishment of cell lines from the xenografts. These PDX tumors were histologically and genetically similar to the patients from where they originated and to xenografts reestablished from cell lines in culture. Most recently, a Danish research group successfully established three SCCA PDX models (unpublished, presented at IMAAC2023). Invasive analysis of administered hypoxia probes and hypoxia-regulated genes in an HPV-positive and HPV-negative model revealed that both were profoundly hypoxic, which was in agreement with the patient tumors from where they originated (Figure 4).^12^ In contrast, there are at least 61 cervical PDX models established.^101^

**Figure 5:**
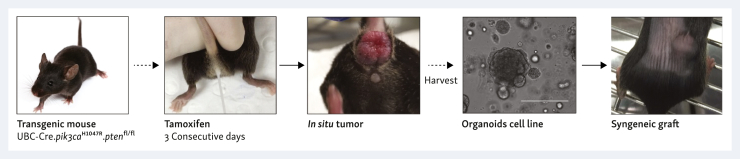
**Development of SCCA following targeted 4-OHT application to the anal canal in UBC-Cre.*pik3ca*^*H1047R*^*.pten*^*fl/fl*^C57Bl/6 mice.** The subsequent tumor was then harvested for the development of a mouse SCCA cell line, which can be implanted as a syngraft in wtC57Bl/6 mice. 4-OHT, 4-hydroxytamoxifen; SCCA, squamous cell carcinoma of the anus.

Another SCCA mouse model in Australia has been established, based on a transgenic C57Bl/6 mouse with a tamoxifen-inducible ubiquitin C-Cre-conditional knock-in of a *Pik3ca*^*H1047R*^ mutation and double deletion of *PTEN* (UBC-Cre.*pik3ca*^H1047R^.*pten*^fl/fl^ C57Bl/6) (Figure 5) (unpublished). Targeted application of 4-hydroxy-tamoxifen to the anal canal leads to the development of tumors. The tumors were confirmed to be invasive SCC and arising from the anal epithelium on histopathology. A syngeneic cell line has also been established from an SCCA of this mouse, with morphological and histological features consistent with SCCA. This line was transduced with human HPV 16 E6 and E7 oncogenes to create a separate line that recapitulated an HPV-positive mouse SCCA. Both lines form tumoroids in extracellular matrix, with similar histology to comparative human SCCA cell line tumoroids. They also demonstrate tumorigenicity in immunocompromised NOD scid gamma and syngeneic wtC57Bl/6 mice, including growing orthotopically within the anal canal. A sub-clone of the E6/7 line has metastatic potential, with pulmonary lesions confirmed to be SCC on histology. Therapeutic assays revealed the mouse lines to be more sensitive to 5-fluorouracil but with a similar response profile to mitomycin-C as the human lines and greater resistance to RT. Assessment by flow cytometry of the immune infiltrate in the SCCA mouse cell line syngrafts demonstrated many similarities with the immune phenotype of human SCCA. A co-culture cytotoxic assay with syngraft TILs and mouse SCCA cell line tumoroids demonstrated limited killing, similar to the two human SCCA tumoroid lines developed from patients with relapsed disease.

## Conclusion

Similarities in treatment regimens, overall outcome, and biology between the different HPV-associated SCCs allow for the extrapolation of many observations generated from one SCC tumor type to another. There is still an urgent need, however, to generate a deeper understanding of specific SCCA biology, and the present overview focused on progress in SCCA research in the context of other SCCs. An international network has been formed to exchange knowledge and collaborate on preclinical and translational studies in this rare disease, which is mandatory for the much-needed development.
